# Border analysis for spatial clusters

**DOI:** 10.1186/s12942-018-0124-1

**Published:** 2018-02-17

**Authors:** Fernando L. P. Oliveira, André L. F. Cançado, Gustavo de Souza, Gladston J. P. Moreira, Martin Kulldorff

**Affiliations:** 1 Department of Statistics, UFOP, Morro do Cruzeiro, Campus Universitário, Ouro Preto, MG 35400-000 Brazil; 2 Department of Statistics, UnB, Brasília, DF Brazil; 3 Department of Mathematics, UFOP, Ouro Preto, MG Brazil; 4 Department of Computer Science, UFOP, Ouro Preto, MG Brazil; 5 Division of Pharmacoepidemiology and Pharmacoeconomics, Harvard Medical School, Boston, MA USA

**Keywords:** Spatial scan statistic, Border analysis, Cluster delineation, Disease surveillance, Disease mapping

## Abstract

**Background:**

The spatial scan statistic is widely used by public health professionals in the detection of spatial clusters in inhomogeneous point process. The most popular version of the spatial scan statistic uses a circular-shaped scanning window. Several other variants, using other parametric or non-parametric shapes, are also available. However, none of them offer information about the uncertainty on the borders of the detected clusters.

**Method:**

We propose a new method to evaluate uncertainty on the boundaries of spatial clusters identified through the spatial scan statistic for Poisson data. For each spatial data location *i*, a function *F*(*i*) is calculated. While not a probability, this function takes values in the [0, 1] interval, with a higher value indicating more evidence that the location belongs to the true cluster.

**Results:**

Through a set of simulation studies, we show that the *F* function provides a way to define, measure and visualize the certainty or uncertainty of each specific location belonging to the true cluster. The method can be applied whether there are one or multiple detected clusters on the map. We illustrate the new method on a data set concerning Chagas disease in Minas Gerais, Brazil.

**Conclusions:**

The higher the intensity given to an area, the higher the plausibility of that particular area to belong to the true cluster in case it exists. This way, the *F* function provides information from which the public health practitioner can perform a border analysis of the detected spatial scan statistic clusters. We have implemented and illustrated the border analysis *F* function in the context of the circular spatial scan statistic for spatially aggregated Poisson data. The definition is clearly independent of both the shape of the scanning window and the probability model under which the data is generated. To make the new method widely available to users, it has been implemented in the freely available SaTScan$^\mathrm{TM}$ software www.satscan.org.

## Background

Spatial statistical analysis is commonly used by epidemiologists and public health professionals to study geographical disease patterns. For instance, in disease surveillance, spatial cluster detection techniques are often used to detect areas with excess disease incidence, prevalence or mortality [1–10]. Different types of spatial scan statistics are the most commonly used methods for the detection and statistical inference of spatial disease clusters [11–26]. They have been used in many different locations for a wide variety of diseases [10, 27]. For example, they have been used to analyze the geographical distribution of Dengue fever in Guangdong Province [28], malaria hot spots in Kenya [29], the geography of bovine tuberculosis in the Madrid region [30] and the spatial variation inpost-injury quality of life outcomes in Vancouver [31] and gastric cancer [32].

The key feature of all spatial scan statistics is that they adjust for the multiple testing inherent in the many potential cluster locations and sizes that are evaluated. Equally important, they can adjust for heterogeneous population densities as well as any number of confounding variables such as age or socio-economic factors. Moreover, most spatial scan statistics are applicable to point data as well as to aggregate data.

One limitation of the spatial scan statistic is that it is not possible to determine the exact borders of the detected clusters. While the spatial scan statistic will generate a well-defined most likely cluster, which is the cluster that is least likely to have occurred by chance, there are almost always many highly overlapping clusters of almost the same magnitude or strength. That is, if the most likely cluster is statistically significant, there will be many other statistically significant clusters with only slightly different boundaries, and with a p-value that is almost as small. The reason for this is that moving the scan window slightly so that a few additional locations get included or excluded from the cluster will not change the likelihood function very much in either direction.

When presenting the results from a spatial scan statistic analysis on a map, one option is to show all the statistically significant clusters. With hundreds or thousands of such highly overlapping clusters, such a map creates more confusion than clarity, and many of those clusters are significant just because a small part of the cluster has a large number of disease cases, while other parts of the cluster could be empty. A better approach is to present a limited collection of some of the clusters with the highest likelihood.

One key question is how to decide what collection of clusters to present as part of the results and/or visualize on a map. For example, is it better to present two small non-overlapping clusters or one larger cluster that contains the two smaller ones? The standard approach is to present the most likely cluster as well as any secondary clusters that do not overlap with the more likely present cluster. Boscoe et al. [33] and Chen et al. [34] proposed using not only the likelihood and statistical significance, but also the observed relative risk when determining which clusters to depict on the map. A newer alternative approach is to use the Gini coefficient to determine an appropriate collection of non-overlapping clusters to present [35].

While it is normally advisable to only report non-overlapping clusters, or at most a few overlapping ones, this can lead to a false sense of certainty with respect to the cluster borders. A key question is then whether the reported cluster boundaries are reliable or not. None of the approaches mentioned above will provide any direct information about the uncertainty in the cluster borders. Are all the locations within the detected cluster equally likely to belong to the true cluster? Are there some areas outside the detected cluster that could potentially be part of the true cluster? A true cluster is an area for which the underlying risk of the disease is higher when compared to the risk outside it [15]. Boundary questions are of course relevant for many different types of spatial analyses, and the issue has been well studied outside the realm of scan statistics [36–39]. For example, Goovaerts et al. [40] proposed a criterion to measure the uncertainty of each area being part of a putative spatial cluster.

For spatial scan statistics, one option for studying the cluster boundaries is to use an elliptic or non-parametric spatial scan statistic rather than the circular version. The circular spatial scan statistic has some limitations concerning the detection of irregularly shaped clusters [41], and in such cases, the circular scan statistic can never perfectly delineate the true borders of the cluster. While an irregular shaped cluster will typically have a higher likelihood, their boundaries may not necessarily more accurate [41].

The first attempt to evaluate the uncertainty in the specification of spatial clusters calculated an intensity function for Oliveira et al. [42]. For each data location on the map taking values in the range [0,1]. A higher value means more confidence that the location is in the true cluster. If there is more than one true cluster, the value reflects the confidence that it is in the primary cluster. The details of the intensity function are described in "Spatial scan statistic for Poisson data" section of this paper. For now, we just want to point out that the method tends to propose many locations outside the detected most likely cluster that, maybe, should be part of the cluster, while seldom proposing that any location inside the cluster does not belong there.

In this paper, we propose an alternative to the *intensity function*, which we call the *F* *function*. Like the former, the *F* function is calculated for each data location on the map, taking values in the [0,1] range. It is a more refined technique though, in two ways. Firstly, it will suggest part of the detected cluster that may be less likely to belong to the true cluster, as well as locations outside the detected cluster that could potentially be part of the true cluster. In effect, it gives a fuzzy cluster boundary with uncertainty on both sides of the detected cluster boundary. Secondly, unlike the *intensity function*, it provides a boundary analysis for both the primary and any number of secondary clusters detected. Such information provided by the *F* function can be highly valuable, for instance, in epidemiological cluster investigations, where a public health professional may wish to expand outside the detected cluster area to examine additional observed cases. In this case, the F function can help to decide in what directions to expand the investigation.

In a simulation study with known true clusters, we show that on average, the *F* function performs very well in depicting the uncertainty in the cluster boundaries. We also illustrate the method on a real data set, looking at Chagas disease in Minas Gerais, Brazil. The new method has been implemented in the free and user friendly $\mathrm {SaTScan}^{\mathrm {TM}}$ software (www.satscan.org), so that it can be easily used by the interested reader and public health practitoners.

The rest of this paper is organized as follows. In "Spatial scan statistic for Poisson data" section we offer a brief review of the spatial scan statistic for discrete Poisson data. In "The* F* function for clusters boundary uncertainty" section we present the new method for boundary evaluation, using the [0,1]-valued *F* function to represent the uncertainty in the boundary of the spatial scan statistic clusters detected. Section "A simulation study" presents a simulation study conducted with artificially generated data, using known true clusters. A real data application is presented in "Example: chagas disease in newborns in Minas Gerais" section, looking at Chagas disease in Minas Gerais. Finally, some remarks and topics for future research are discussed in "Discussion" section.

## Spatial scan statistic for Poisson data

While the principles of the new border analysis described in this paper can be applied to many different types of scan statistics, we will consider its implementation for the circular purely spatial scan statistic for spatially aggregated Poisson data. For the sake of completeness, we briefly review that method here.

For the spatial scan statistic, statistical inference for the detected clusters is performed through a likelihood ratio test, as follows [11]. Assume that we have a collection of data locations across a map, indexed by $i=1,\ldots ,K$. For example, these may be postal-code areas or census tracts. Next, consider a set *Z* of a large number of zones generated by considering different collection of neighboring locations. Kulldorff [11] does not make any assumption regarding the shape of the zones, but the set of zones is commonly the collection of all circles that are centered on one of the data locations, with some upper limit on their size. The set of zones could also be a different collection of circles, a collection of ellipses [16], or a collection of irregular shaped zones created in a variety of ways [13–15, 17, 24, 41].

For each location *i*, the number of disease cases $c_i$ follows a Poisson distribution with $p_i*n_i$ expected cases, where $n_i$ represents the population or covariate adjusted population in location *i*, and $N=\sum _{i=1}^{K} n_i$. Under the null hypothesis, $p_i=p$ for all *i*. Under the alternative hypothesis, there is a zone $z \in Z$, such that $p_i=p$ for all $i \in z$, while $p_i=q$ for all $i \notin z$, with $p>q$. The test is conditioned on the total number of cases $C=\sum _{i=1}^{K}c_i$.

It can be shown that the likelihood function for the above is

1 $$\begin{aligned} L(z,p,q) = \dfrac{e^{-pn_z -q(N-n_z )} }{C!} p^{c_z }q^{C- c_z }\left( \displaystyle \prod _{i}n_i \right) . \end{aligned}$$

The likelihood ratio test statistic is given by

2 $$\begin{aligned} \lambda = {\displaystyle \sup _{z\in Z, p>q } } \dfrac{L(z,p,q) }{L_0 },\; p,q\in [0.1], \end{aligned}$$

where $L_0$ is the likelihood function under the null hypothesis

$$\begin{aligned} L_0 = \dfrac{e^{-C } }{C! }\left( \dfrac{C}{N} \right) ^{C} \left( \displaystyle \prod _{i}n_i \right) . \end{aligned}$$

Notice that the test statistic is maximized over all zones $z \in Z$, thus identifying the zone that constitutes the most likely cluster. This is the cluster that is least likely to have occurred by chance.

As is customary we will use the log-likelihood ratio $LLR=\log \lambda$ instead of $\lambda$ as the test statistic.

There is no closed form expression of the distribution of the spatial scan test statistic $\lambda$. The statistical significance of the most likely cluster of observed cases is instead computed through Monte Carlo hypothesis testing [43]. This is done by generating a large number, say $M=999$, of random data sets generated under the null hypothesis, conditioned on the total number of observed cases. The use of $M=999$ is a standard choice in the literature [44–47]. Inference will be more accurate as *M* increases and $M=999$ offers a good trade off between accuracy and computational effort. The test statistic is then calculated for each of those 999 random data sets, and these are compared to the test statistic from the real data set. If the latter is among the highest $5\%$, then it is significant at the $\alpha =0.05$ level. The Monte Carlo based p-value is $p=R/(M+1)$, where *R* is the rank of the test statistic from the real data set (see, for instance, [48]).

## The *F* function for clusters boundary uncertainty

When the spatial scan statistic detect a cluster, we can trust the general location and size of the cluster, but there is always uncertainty in the exact border. Some areas inside the detected cluster may not be part of the true cluster, while some areas outside the detected cluster could be part of the true cluster. One reason for the discrepancy can be that the shape of the true cluster is not one of the cluster shapes used by the spatial scan statistic, but that is not the only reason. Even if the true cluster is among the scanning windows used, parts of the true cluster will have fewer cases than expected, and if they are on the border of the cluster, they may fall outside the detected cluster. Likewise, some areas outside the true cluster will by chance have more cases than expected, and if these cases happen to be just outside the true cluster they may be included in the detected cluster. While it is not possible to determine the true cluster borders with certainty, it is possible to provide some information regarding the uncertainty in the cluster boundary, by using the *F* function that will now be described.

The basic premise of the *F* function is the following. For each data location or administrative area *i*, such as postal-code areas or census tracts, the *F*(*i*) function will take a value in the range [0,1]. A higher value means that there is more evidence that the location is part of the true cluster, while a lower value means that there is less evidence. When $F(i)=0$, there is no evidence that the location is part of the true cluster. While the *F* function only takes values in the [0,1] range, it should be interpreted as a fuzzy possibility.

The first step when calculating the *F* function is to generate *M* randomly drawn bootstrap replicates of the case data. Conditioned on the total number of cases, this is done in the same way as one generate the random data sets for Monte Carlo hypothesis testing, except that data is not generated under the null hypothesis. Instead, the observed counts in the real data set are used as the expected counts when generating the random data sets. This means that an area with an excess number of cases in the real data set will generally also have an excess number of cases in the random data sets, but sometimes more and sometimes less than in the real data.

In formal language, for each administrative area *i*, $i=1,\ldots ,K$, the number of simulated cases $s_i$ is randomly distributed with $c_i$ expected counts, where $c_i$ is the observed number of cases in the real data set. Since we condition on the total number of cases, $\sum _{i} s_i = \sum _{i} c_i = C$, and the random data replication is generated by randomly distributing the total number *C* of cases among the *K* areas according to a multinomial distribution, with the probability associated to area *i* to be $c_i/C$. This procedure is repeated *M* times with *M* large, for example 1000.

As the second step, we apply the spatial scan statistic on each of the *M* randomly generated data sets. For each data set $m=1,\ldots ,M$, we find the most likely cluster, which is the one that maximized the log likelihood ratio test statistic described in the previous section.

For each random data set $m=1,\ldots ,M$ construct the binary *K*-dimensional row vector $MLC_m$ with the *i*-th entry $\left( MLC_m\right) _i$ defined by

$$\begin{aligned} \left( MLC_m \right) _i&= {\left\{ \begin{array}{ll} 1, \text{ if } \text{ location } \text{ i } \text{ belongs } \text{ to } \text{ the } \text{ most } \text{ likely } \text{ cluster }\\ 0, \text{ otherwise } \end{array}\right. }, \end{aligned}$$

and consider the $M\times K$ matrix $MLC=\left[ MCL_1 \ldots MCL_M\right] ^{T}$. That is, $MLC=\left( MLC_{ij}\right) _{m\times K}$ with $\left( MLC_m\right) _i =1$ if the most likely cluster found in the *m*-th iteration contains region *i*, and $MLC_{mi}=0$ otherwise.

As the third step, we define the *F* *function* as the real-valued function $F:\{ 1,\ldots ,K \}\longrightarrow [0,1]$ giving for region *i* the value

3 $$\begin{aligned} F(i)=\frac{1}{M}\displaystyle \sum _{m=1}^{M} (MLC_m)_i,\; i=1,\ldots ,K. \end{aligned}$$

It is clear from Eq. (3) that the *F* function is defined throughout the whole geographic region under analysis and that it takes values in the interval [0, 1].

In summary, the procedure consists of the following three steps:

1. Conditioned on the total number of cases, a set of *M* random data set is generated. The expected number of cases in location *i* is set equal to the observed number of cases in that location, using a multinomial distribution.
2. The spatial scan statistic is applied to each of the randomly generated data sets with the original population based expected counts. For each random data set, note which of the locations *i* belong to the most likely cluster.
3. For each location *i*, find how many of the *M* most likely clusters contain location *i*. The *F*(*i*) function is defined as the proportion of those most likely clusters that contain location *i*.

### Extension to multiple detected clusters

Above we have defined the *F* function for the special case when there was only one cluster detected in the data. In many situations, there are multiple non-overlapping clusters found in different areas of the map. When multiple non-overlapping clusters have been detected, the *F* function will be defined in the same way with one exception. Suppose there were *D* non-overlapping clusters found in the real data set. In the second step, we will not only find the most likely cluster, but the *D* most likely non-overlapping clusters, using exactly the same procedure as for the real data set. The *i*-th entry of the vector $MLC_m$ will then be equal to one if location *i* is in any of the *D* most likely non-overlapping clusters. Since non-overlapping clusters can be defined in different ways, it is important that the same definition is used when finding the collection of non-overlapping clusters in the real and random data sets.

### Comparison with the intensity function

Compared to the *F* function, the previously proposed intensity function [42] is similar in one way and very different in another way. The similarity is that the *M* bootstrap data sets are generated in exactly the same way, using the observed counts from the real data as the expected counts for the simulated data sets. They are also the same in the way that the spatial scan statistic is applied to the random data sets. The big difference is how the function is calculated.

The intensity function is calculated as follows. For each of the *M* simulated data sets, the most likely cluster is found, that is, the cluster with the maximum log likelihood ratio (LLR) test statistic. These maximum LLRs are then ranked from low to high. Together, they form a set of *M* ranked log likelihood maxima. As the next step, one takes each location *i* in turn, and finds the collection of random data sets in which location *i* belongs to the most likely cluster. Among this collection, we find the one with the highest log likelihood ratio test statistic, and we note its rank. If the rank is *R*, then the intensity function is defined as $q(i)=R/M$ (for details see Oliveira et al. [42]).

Here are five examples of the intensity function *q*(*i*), and how it compares with the *F*(*i*) function.

(A) If location *i* is part of the most likely cluster in all the random data sets, then it is obviously part of the one that generated the highest LLR test statistic, so the rank is *M*, and $q(i)=M/M=1$. We also have $F(i)=M/M=1$.
(B) If location *i* was a member of the most likely cluster in only one of the simulated data sets, and if the maximum LLR test statistic from that data set was higher than from any of the other simulated data sets, then $R=M$ and $q(i)=M/M=1$. As a contrast, $F(i)=1/M$.
(C) If location *i* was a member of the most likely cluster in only one of the simulated data sets, and if the maximum LLR test statistic from that data set was higher than exactly half of the other simulated data sets, then $R=M/2$ and $q(i)=(M/2)/M=1/2$. As a comparison, $F(i)=1/M$.
(D) If location *i* was a member of the most likely cluster in only one of the simulated data sets, and if the maximum LLR test statistic from that data set was lower than from any of the other simulated data sets, then $R=1$ and $q(i)=1/M$. Here we also have $F(i)=1/M$.
(E) If location *i* is not part of the most likely cluster in any of the simulated data sets, then both $q(i)=0$ and $F(i)=0$.

Note that, by definition, $q(i) \ge F(i)$ for all *i*. Note also that $q(i)=0$ if and only if $F(i)=0$.

Notice that while *F* values are frequencies and thus evaluate statistical significance, *q* values rank likelihood ratios and thus evaluate how strong the cluster is.

## A simulation study

We evaluated the performance of the *F* function using artificial data, where we know what the true cluster looks like. Five scenarios containing different true clusters were created: a small circular cluster, a large circular cluster, two small circular clusters, an irregular L-shaped cluster and an elliptic cluster.

All five different scenarios were built over a map which consists of 203 hexagonal cells arranged in a regular grid, each of them with a population of 1000 individuals. For each scenario, $C=20,300$ total cases were then randomly distributed over the map so that individuals in regions inside the cluster are more likely to become cases than in regions outside the cluster. The relative risk inside each cluster was computed so that the power of detection would be 0.99 if we knew the exact location of the cluster in advance, using a significance level of 0.05 [49].

For each true cluster model, $N=100$ random data sets are generated under the alternative hypothesis with an excess relative risk in the true cluster. The relative risk for regions inside each artificial true cluster are computed so that the random distribution of cases will produce a significant cluster formed by those regions with probability 0.99 for high intensity clusters. For each random data set, we found the most likely cluster using the circular spatial scan statistic and we computed the *F* function using $M=100$ bootstrap samples. We then compare the average detected cluster and the average *F* function obtained for the *N* random data set.

**Fig. 1:**
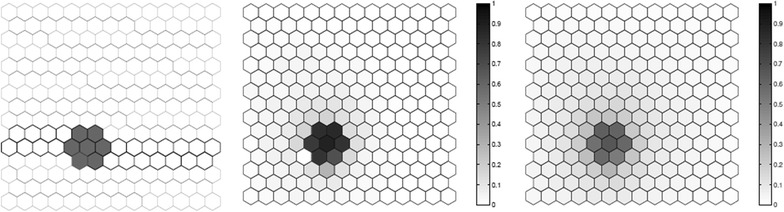
A small circular cluster (left), the average detected cluster (center) and the average *F* function (right)

**Fig. 2:**
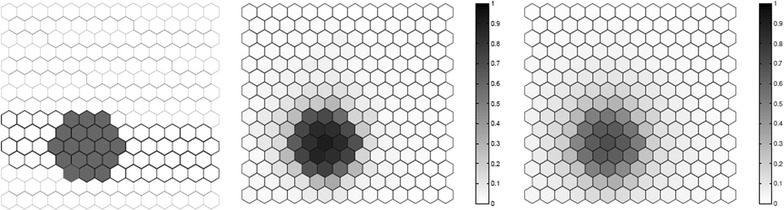
A large circular cluster (left), the average detected cluster (center) and the average *F* function (right)

**Fig. 3:**
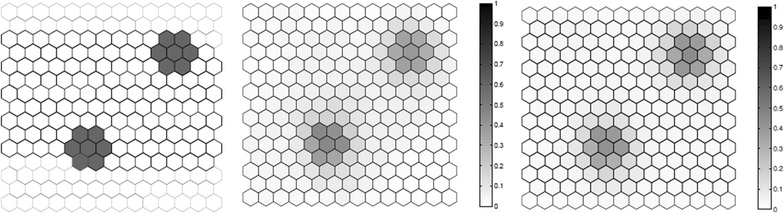
Two clusters (left), the average detected clusters (center) and the average *F* function (right)

**Fig. 4:**
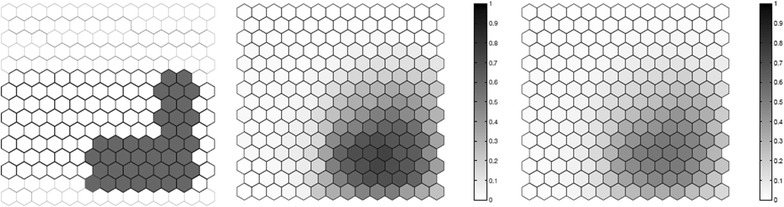
One irregular L-shaped cluster (left), the average detected cluster (center) and the average *F* function (right)

**Fig. 5:**
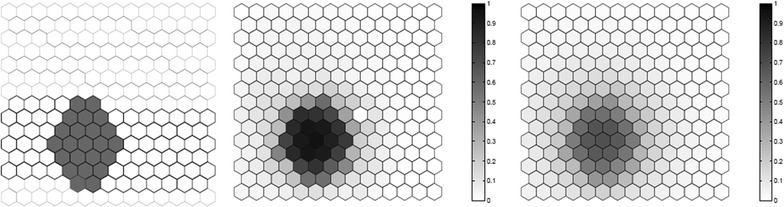
A true elliptic cluster (left), the average detected cluster (center) and the average *F* function (right)

The results for the five different cluster models are shown in Figs. 1, 2, 3, 4 and 5. From these graphs we can see that the average results obtained through the *F* function do not markedly differ from the average most likely clusters. As one would expect, the *F* function is sometimes positive for some locations outside the true cluster, but the average *F* values tend to be lower even if close to the true cluster, and zero or very close to zero for locations further away from the cluster. Moreover, most locations within the cluster tend to have a fairly high *F* value, although never exactly equal to the maximum value $F=1$. The exceptions are the top of the L-shaped clusters and the top and bottom of the elliptic cluster, where there are a few locations within the true cluster that have fairly low *F* values. The worst performance for the L-shaped cluster is showed in Fig. 4. This has less to do with the nature of the *F* function, but is driven by the fact that a circular scan statistic was used to detect a very non-circular cluster. We can see that the average detected cluster often fails to detect the extreme edges of the L-shaped cluster.

Ideally, a “perfect” method should result in a value equal to 1 for locations belonging to the true cluster and zero for locations outside of it. For comparison purposes, consider $t(i) = 1$ if the *i*-th location belongs to the true cluster, and $t(i) = 0$ otherwise. Let $F_n (i)$ be the value of *F* for the *i*-th location for the *n*-th random data set, and let $MLC_n(i) = 1$ if the *i*-th location belongs to the most likely cluster for the *n*-th random data set, and equal to zero otherwise. Then, we can calculate the Euclidean distance between the true cluster given by *t* and its estimates given by the $F_n$ function and the most likely cluster $MLC_n$ for the *n*-th data set, respectively, by

$$\begin{aligned} {d (F_n, t) = \sqrt{\sum _{i = 1}^K (F_n(i)-t(i))^2)}} \end{aligned}$$

and

$$\begin{aligned} {d (MLC_n, t) = \sqrt{\sum _{i = 1}^K \left( MLC_n(i)-t(i))^2\right) }}. \end{aligned}$$

Table 1 lists the values of the average Euclidean distances $\overline{d}(F_n,t)=\sum _{n=1}^{N} d(F_n,t)/N$ and $\overline{d}(MLC_n,t)=\sum _{n=1}^{N} d(MLC_n,t)/N$ for the $N=100$ random data sets. From Table 1 it is clear that the *F* function is systematically closer to the true cluster than the most likely cluster.

**Table 1:** Average distances from the true cluster to the *F* function and to the most likely cluster

Scenario	$\overline{d}(F_n,t)$	$\overline{d}(MLC_n,t)$
Small	1.77	1.91
Large	2.72	3.08
Double	2.81	3.49
Irregular	4.61	5.83
Elliptic	3.09	3.64

## Example: chagas disease in newborns in Minas Gerais

To illustrate with real data, the new boundary analysis method was applied to Chagas disease cases in new-born babies in the state of Minas Gerais, Brazil. Chagas disease is a parasitic disease caused by *Trypanosoma cruzi*. The vertical mode of transmission, from mother to child, is considered one of the main routes of transmission of the disease. In Brazil, more than 3 million people are affected by Chagas disease, and more than 600,000 of these reside in the state of Minas Gerais.

The population at risk consists of all children born in Minas Gerais, from July to September 2006. The new-born children were blood tested to detect the presence of the Chagas disease antigen, with coverage above 96%. The tests were conducted through the Minas Gerais State Program for New-Born Screening (PETN-MG), coordinated by the NUPAD-MEDICINA/UFMG research group from the Federal University of Minas Gerais Medical School, in collaboration with the Minas Gerais State Health Secretary. The data was previously analyzed by Oliveira et al. [42].

**Fig. 6:**
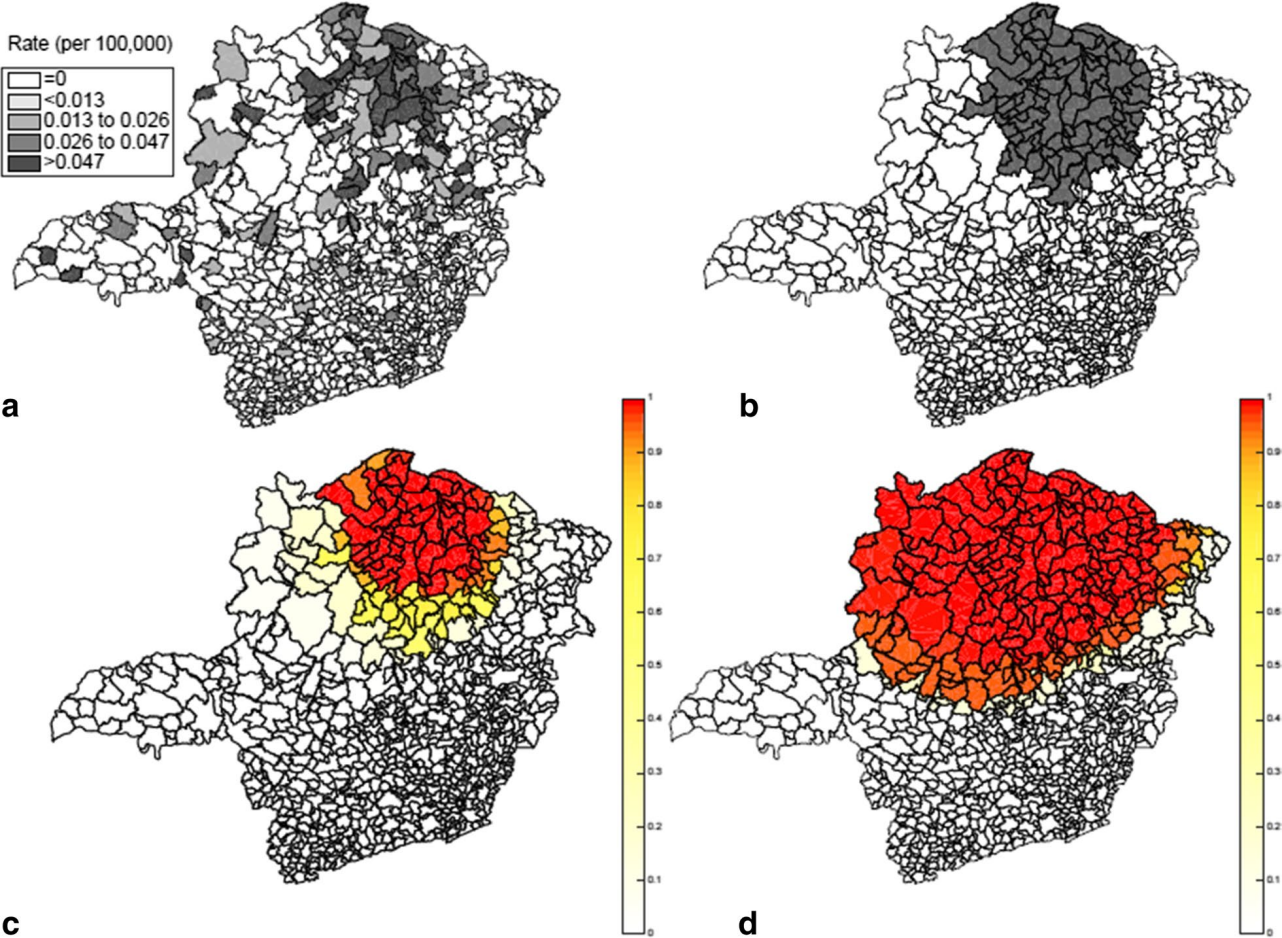
Chagas disease rates in newborn babies (**a**), the most likely cluster detected by the circular spatial scan statistic (**b**), boundary analysis obtained by the *F* function (**c**) and intensity function (**d**)

The state is divided into 853 municipalities with a total population at risk of 63,519 new-born babies. A comprehensive screening to eliminate false positives was done. The data set consists of a pair of coordinates (*x*, *y*), the number of cases and the population at risk for each of the 853 municipalities. To analyze the data, we used the Poisson based spatial scan statistic with a circular scanning window. The maximum cluster size allowed was 50% of total population. Only one statistically significant cluster was found. Located in the northern part of the state, it had 528 Chagas disease cases among new-borns, a relative risk of 5.09, with $p< 10^{-17}$. In Fig. 6, the rate of Chagas disease in each municipality is shown on the top left side while the detected cluster is shown on the top right.

The result of the *F* function boundary analysis is shown in Fig. 6. The area formed by the highest *F* values coincides well with the primary cluster found by the circular scan statistics showing a presence of a strong anomaly. It is only the southern parts of the detected cluster that have somewhat lower *F* values, indicating some uncertainty as to whether this southern part belongs to the true cluster. Regarding the areas outside the detected cluster, the locations to the west have low but not insignificant low *F* values. Hence, there is some weak evidence that these areas to the west may belong to the true cluster. In general, this example shows that it was possible to evaluate and visualize the uncertainty of the borders of the detected cluster by means of the *F* function boundary analysis.

The result of applying the intensity function on the same Chagas disease data is shown in the bottom right part (d) of Fig. 6. We observe that the potential cluster area is less geographically focused with a much wider spread than for the *F* function. As special note is that all of the location of the detected cluster has a very high intensity, at or close to one. This means that the intensity function has a hard time to determine which areas within the detected cluster may not truly belong to the true cluster.

## Discussion

The spatial scan statistic is a commonly used method for detecting and evaluating the statistical significance of spatial disease clusters, but there is always uncertainty in the actual borders of the true underlying cluster. We have proposed the boundary analysis *F* function as a tool to evaluate what areas are more or less likely to belong to the true underlying cluster.

Intuitively, one can think of the *F* function as giving a cluster intensity function on the study region of interest, assigning to each area a normalized intensity value in [0, 1] range. The higher the intensity given to an area, the higher the plausibility of that particular area to belong to the true cluster in case it exists. Through the bootstrap procedure, instead of obtaining a single point estimate of the cluster we were able to obtain a collection of “measures” that approximate the true cluster in a more stable way, on average. While the estimation based only on the observed data can detect a cluster slightly different from the true one by chance, the average of the estimates obtained via bootstrap should assign high values of the *F* function to regions that belong to the true cluster, and smaller for regions that do not belong to it, since that specific error is unlikely to be repeated over most replications. Besides, even though the shapes of the scan window and the true cluster mismatch, since we measure the cluster several times by means of the bootstrap approach, the final composition would still be able to approximately reproduce the actual cluster, on average. In this manner, the *F* function provides information from which the public health practitioner can perform a border analysis of the detected spatial scan statistic clusters. For instance, in a cluster investigation, a public health professional may wish to expand outside the detected cluster area to examine additional observed cases, and the *F* function can then help decide in what directions to expand the investigation.

In this paper, we have implemented and illustrated the border analysis *F* function in the context of the circular spatial scan statistic for spatially aggregated Poisson data. The definition is clearly independent of both the shape of the scanning window and the probability model under which the data is generated. Hence, it could easily be adapted and applied to the elliptic or irregular shaped spatial scan statistics, or, scan statistics for Bernoulli, multinomial, normal, exponential data. Of course, how it will perform in practice for such data needs further investigation. It is also unclear how the *F* function will perform for less aggregated data, where there is at most one disease case in the majority of the locations. Lastly, it would be very interesting to develop a modified *F* function for use with space-time scan statistics.

The *F* function, as described in this paper, is computer intensive, as it requires the generation of multiple random data sets. To compute the *F* function we need to apply the scan statistic on each of the $M=999$ randomly generated data sets. Thus, the computational effort is exactly the same needed to perform the $M=999$ Monte Carlo simulations under the null hypothesis to compute the significance of clusters detected in the standard approach. To make the new method widely available to users, it has been implemented in the freely available $\mathrm {SaTScan}^{\mathrm {TM}}$ software www.satscan.org.
